# Current questions and controversies in chromosome fragile site research: does *WWOX*, the gene product of common fragile site FRA16D, have a passive or active role in cancer?

**DOI:** 10.1038/cddiscovery.2015.40

**Published:** 2015-10-19

**Authors:** I Hazan, RI Aqeilan

**Affiliations:** 1 Lautenberg Center for Immunology and Cancer Research, IMRIC, Hebrew University-Hadassah Medical School, Jerusalem, Israel

- The controversy about common fragile site (CFS) and their associated genes/products is whether their aberrations in cancer passively accumulate or actively contribute to cancer development.
- The WW domain-containing oxidoreductase (*W* *WOX*) gene spans one of the most active CFS FRA16D and is commonly altered in cancer.
- *WWOX* encodes a tumor suppressor that enhances efficient DNA damage response (DDR) functions ranging from DNA repair and apoptosis to suppression of tumorigenic signaling pathways.
- *WWOX* deficiency results in cancer progression not only because of the fragility but also because it is enriched by selective pressure during tumorigenesis.

The role of CFSs in cancer is debatable and two major views dominate the discussion in this regard. One view suggests that CFSs are hotspots for genomic instability leading to damage of genes residing within them and hence losing these genes is unselected 'passenger' or ‘passive’ event. The counter argument is that losing these genes leads to selective pressure as a result of their tumor-suppressive activities and therefore directly contributes to carcinogenesis and have an active role: a ‘driver’ event.

Genomic instability, one of cancer hallmarks, is characterized by mutations, deletions, amplifications and translocations.^1^ In particular, we focus here on genomic aberrations related to large deletions since CFSs have been known for decades as ‘hotspots’ for deletions and breaks taking place in metaphase chromosomes as a result of replication stress.^2^ Indeed, the most significant deletions in human tumor specimens and cancer cell lines coincide with CFSs or large genes.^3–5^ Many of these deletions are hemizygous, suggesting, although not proven, that the other allele is maintained. This assumption led to the hypothesis that alterations in CFSs are secondary events that do not contribute to the multistep tumorigenesis process. However, emerging evidence suggests elsewise as CFSs do not seem to be inert structures but contain functional elements, such as tumor suppressor genes, coding and noncoding, that have critical roles in carcinogenesis.^6^

One such example of a tumor suppressor gene is the WW domain-containing oxidoreductase (*WWOX*), which spans the CFS FRA16D. *WWOX* is among the most significantly deleted genes in cancers,^3,4,7^ suggesting a selective pressure because of a tumor suppressor activity and also because of the fragility of that locus. Accordingly, emerging findings demonstrate that WWOX protein has pleiotropic tumor-suppressive functions, particularly by regulating the DNA DDR.^8^

The *WWOX/FRA16D* locus, similar to other CFS genes, is an evolutionarily conserved, megabase long that is predisposed to DNA single- and double-strand breaks (SSBs and DSBs, respectively) in response to intrinsic (oncogenic) or extrinsic (environmental) replication stress. Therefore, *WWOX* and other CFS genes might be an early warning sensor for DNA damage. Because of their sensitivity, they are prone to allelic imbalances and deletions associated with genomic instability and mutator phenotype that further drive tumor progression. Here we discuss supporting evidence favoring active and tumor suppressor functions of *WWOX* in tumorigenesis.

## Alterations of *WWOX* in cancers support its role as a tumor suppressor

*WWOX* was originally cloned as a putative tumor suppressor because of its frequent loss in cancer (reviewed in Gardenswartz and Aqeilan^7^). Data obtained from the TCGA copy number portal demonstrated that the *WWOX* locus is the fifth most frequently deleted locus in 10 844 samples from 33 cancer types (Q-value=1.31E^−266^; http://www.broadinstitute.org/tcga/home). Well-known tumor suppressors such as *PTEN*, *RB1* and *TP53* are significantly deleted (0, 7.24E^−273^ and 5.04E^−22^, respectively), although the deleted regions have additional genes within their peaks (i.e. *PTEN* with the tumor suppressor *KLLN*); *WWOX*, on the other hand, is a single gene within the deleted peak emphasizing that losing its own activity contributes to cancer. In addition, translocations within this region (14q32;16q23) were observed in up to 25% of multiple myelomas.^7^

Loss of *WWOX* is associated with poor prognosis in numerous cancers and may result not only from deletions and translocations but also from epigenetic silencing by DNA methylation or mutations within its promoter.^7^

Another interesting observation is that most of the deletions within *WWOX* are hemizygous rather than homozygous, suggesting haploinsufficiency. In support of this notion, *Wwox* heterozygous mice develop higher incidence of spontaneous tumors.^9^ Furthermore, McAvoy *et al* ^5^ reported that there is no relationship between alteration of a given CFS and expression of its associated gene product in a given cancer, suggesting that these alterations are not merely responsible for dysregulation of WWOX protein expression. It is therefore possible that at early stages of tumorigenesis, breaks in FRA16D alert the DDR to warn against environmental or intrinsic cues. But is this the end of the story or that a following chapter do exist?

## *WWOX* functions as a tumor suppressor

Regardless of *WWOX* localization in the genome and causes of its aberration in cancer, emerging findings indicate that WWOX tumor-suppressive functions range from involvement in DDR to modulation of tumorigenic signaling such as HIF1*α*, TGF*β*/SMAD and WNT/*β* catenin (reviewed in Aqelian *et al*.^10^).

## *WWOX* facilitates DDR

Although *WWOX* locus is vulnerable to DNA damage, levels of WWOX protein were recently shown to be induced following DSBs^8^ and SSBs (unpublished data). Upon DSBs, *WWOX* physically interacts and supports activation of the checkpoint kinase ATM allowing efficient and accurate DNA repair.^8^ ATM is activated early after DSBs and phosphorylates the ubiquitin E3 ligase ITCH and other substrates, including H2AX, CHK2 and p53.^8^ ITCH mediates K63-linked ubiquitination of *WWOX* and promotes its translocation into the nucleus where it activates ATM in a positive feed-forward loop manner.^8,^ ^10^ *WWOX* deficiency results in reduced activation of ATM and defects in recruitment of components of the repair machinery to damaged sites,^8^ resulting in increased DSBs. Overall, loss of *WWOX* impaired DNA repair, which may lead to genomic instability and tumorigenesis.^10^

## *WWOX* promotes apoptosis

Ectopic expression of *WWOX* in different cancer cell lines resulted in apoptosis *in vitro* and marked inhibition of tumorigenicity *in vivo*. Cumulative evidence has shown that *WWOX* regulates cell death through interactions with members of the p53 family of proapoptotic transcription factors p53, p73 and p63 through various mechanisms. Although the interaction between *WWOX* and p53 occurs only when phosphorylated WWOX enters the nucleus,^11^ cytoplasmic unphosphorylated WWOX can interact with p73 leading to transactivation-independent apoptosis. WWOX interaction with ΔNp63*α* inhibits its nuclear translocation and suppresses its transactivation function.^12^ In addition, *WWOX* has also been shown to interact with mTOR to suppress autophagy and induce apoptosis and methotrexate sensitivity.^13^

## Conclusions

Tumor-suppressing functions of *WWOX* identify it as a master regulator that protects from genomic instability (but also having major roles in other cellular process that could not be discussed here). Therefore, we propose that *WWOX* loss early in the precancerous stage is not a secondary/passive event because of the fragility of its locus but provides a growth advantage that actively contributes to tumorigenesis. Further studies of WWOX function might shed light on the intriguing question of why one of the most unstable chromosomal regions in the genome, FRA16D, is evolutionarily conserved and harbors a tumor suppressor? One explanation might be that cancer usually occurs after the reproductive phase and therefore loss of CFS would not have an inherited selective pressure. In support of this assumption, germline mutations or loss of function of WWOX are associated with neuronal disorders,^14^ whereas somatic *WWOX* lesions are associated with cancer.^15^

**Figure 1:**
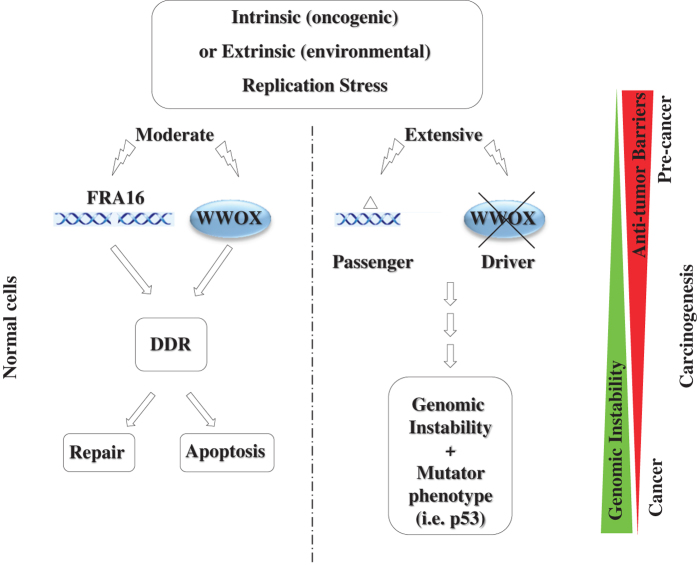
Hierarchical model for WWOX/FRA16D mediating cancer development. Both the fragile site FRA16D and its gene product *WWOX* are affected by replication stress. Although FRA16D is prone to DSBs, WWOX activity is induced resulting in DDR and/or apoptosis. Upon extensive damage, breaks within FRA16D inaccurately repaired as deletions. Cells with deletions within WWOX-encoding gene (depicted as Δ) are positively selected owing to the role of WWOX in DDR leading to genomic instability and mutator phenotype. Additional mutations in other tumor suppressor loci (such as *p53*) would release antitumor barriers leading to cancer development.

Gorgoulis and co-workers^6^ suggested that scattering ‘sensors’ of coding and noncoding elements in CFSs throughout the genome send alarming signals of replication stress. We further propose that as part of the DDR, gene products of CFSs, such as WWOX,^10^ have active roles in repairing the damage or eliminating the ‘bad’ cells by apoptosis. However, when WWOX is lost, the antitumor barriers are gradually lost and aberrations in classical recessive genes accumulate to feed into carcinogenesis (Figure 1). Therefore, placing a guarding gene within CSF provides an advantage as long as the cellular checkpoints are not compromised, whereas failure in these checkpoints (i.e. by additional mutations in *RB1* and *p53*) would lead to tumor initiation and/or progression. Other CFS genes such as *FHIT*, *SPIDR*, *PARK2* and *RORA* have also roles in DDR, with some having established tumor suppressor function.^5,16^ As the expression of these CFSs and their associated genes is cell-type-specific,^17^ it is reasonable to assume that they vary in their behavior from one cancer type to another.

Further delineation of WWOX tumor suppressor functions and other products of CFSs, both *in vitro* and using animal models, should contribute to better understanding of carcinogenesis. Mapping and timing of CFS genes’ inactivation during the neoplastic process, of different tissues, will be crucial to best dissect their contributions and nail their functions.
